# Can weather generation capture precipitation patterns across different climates, spatial scales and under data scarcity?

**DOI:** 10.1038/s41598-017-05822-y

**Published:** 2017-07-14

**Authors:** Korbinian Breinl, Giuliano Di Baldassarre, Marc Girons Lopez, Michael Hagenlocher, Giulia Vico, Anna Rutgersson

**Affiliations:** 1 Department of Earth Sciences, Uppsala University, Villavägen 16, 75236 Uppsala, Sweden; 2 Department of Geography, University of Zurich, Winterthurerstrasse 190, 8057 Zurich, Switzerland; 3 Institute for Environment and Human Security, United Nations University (UNU-EHS), UN Campus, Platz der Vereinten Nationen 1, 53113 Bonn, Germany; 4 Department of Crop Production Ecology, Swedish University of Agricultural Sciences, Ulls väg 16, 75007 Uppsala, Sweden

## Abstract

Stochastic weather generators can generate very long time series of weather patterns, which are indispensable in earth sciences, ecology and climate research. Yet, both their potential and limitations remain largely unclear because past research has typically focused on eclectic case studies at small spatial scales in temperate climates. In addition, stochastic multi-site algorithms are usually not publicly available, making the reproducibility of results difficult. To overcome these limitations, we investigated the performance of the reduced-complexity multi-site precipitation generator TripleM across three different climatic regions in the United States. By resampling observations, we investigated for the first time the performance of a multi-site precipitation generator as a function of the extent of the gauge network and the network density. The definition of the role of the network density provides new insights into the applicability in data-poor contexts. The performance was assessed using nine different statistical metrics with main focus on the inter-annual variability of precipitation and the lengths of dry and wet spells. Among our study regions, our results indicate a more accurate performance in wet temperate climates compared to drier climates. Performance deficits are more marked at larger spatial scales due to the increasing heterogeneity of climatic conditions.

## Introduction

Precipitation is a key component of the water cycle, which in turn affects terrestrial ecosystems, agricultural production and human well-being. Access to long precipitation time series is crucial for many ecological, agricultural or hydrological studies, as well as for public health and climate research^1, 2^. Many regions lack such wealth of data, so that realistic simulations of precipitation patterns are needed. Simulations have to preserve the spatial and temporal dynamics as well as the correlation structures of precipitation patterns and their variability as they are fundamental for impact analyses^3^.

Precipitations patterns can be simulated either with numerical weather prediction models or stochastic algorithms. These methods are complementary and have specific advantages and drawbacks. Numerical weather prediction models include a physical description of the entire atmosphere and its interaction with the land surface, often also including oceans and vegetation, making the simulated fields physically consistent. This however leads to high computational costs and potential limitations in both the number of simulations that can be generated and their spatial resolution. Typically, the feasible spatial resolution is coarser than required for most impact assessments. Moreover, the accuracy of precipitation fields produced by such models can suffer from spatiotemporal and amplitude errors depending on the model physics, dynamics and model configuration^4, 5^.

Stochastic algorithms, in contrast, require considerably less computational effort and can therefore easily provide long time series. Multi-site stochastic precipitation generators are mathematical algorithms for producing synthetic precipitation based on multiple ground observation sites (i.e. precipitation gauges). They can simulate precipitation patterns in space and time similar to the actual observations. Several algorithms exist, often embedded in weather generators for various climate variables. Stochastic precipitation generators can be used for downscaling of numerical weather models and for climate projections^6–15^, flood and drought assessments^16–21^, agricultural studies^22–24^, food security^25, 26^, as well as public^27–29^ and veterinary health^30^. The main drawback of statistical methods is that, while spatial and temporal correlation structures are kept, unlike numerical weather prediction, they cannot simulate the associated large-scale dynamics leading to temperature and precipitation variabilities.

Despite this limitation, there is undoubtedly potential for more extensive application of multi-site precipitation generators. Yet surprisingly little knowledge is available regarding their application across spatial scales, in different climates and under conditions of data scarcity. So far, stochastic multi-site precipitation generators have been primarily applied at small spatial scales (not exceeding some tens of kilometers), with only a handful of sites^10, 13, 31–37^. Only very few authors have focused on larger spatial scales^38–41^. The majority of these studies has been carried out in temperate and precipitation-rich climates in developed countries, where dense observation networks and long time series of reliable climate data are the norm^8, 9, 19, 20, 22, 24, 39, 42, 43^. Furthermore, the widespread application of precipitation generators has been limited by the lack of publicly available transparent source codes and the mathematical complexity of many models, so that setting up a model still requires major efforts. The complexity of algorithms has been recently identified as an issue by Apel *et al*.^44^ and the fragmented body of knowledge has been critically reviewed by Ailliot *et al*.^45^.

Towards an easier and more widespread use of stochastic multi-site precipitation generation, here we first assess the performance of multi-site precipitation generation across three different climatic zones and across spatial scales in the United States, from about thirty kilometers to over one thousand kilometers of maximum extent. Second, we link the density of the observation network to the performance of the precipitation generation, to provide new insights into model performance under conditions of data scarcity and thus into the applicability in data-poor regions, such as in emerging economies and developing countries.

Addressing these multiple aspects requires the generation of very large data amounts that go far beyond what has yet been presented: for our study we generated almost 1.5 million years of synthetic precipitation. For this reason, we use the latest version of the very fast reduced-complexity stochastic multi-site precipitation generator TripleM (Multisite Markov Model), which requires only two key parameters for simulating any gauge network in its simplest setup. Other algorithms require a very large number of parameters that grow exponentially with the number of gauges^42^, making comprehensive studies not feasible. To our knowledge, TripleM is the most straightforward multi-site precipitation generator currently available and thus probably one of very few models that allows for comprehensive studies.

## Data and Experiments

### Station-based climate observations

**Figure 1:**
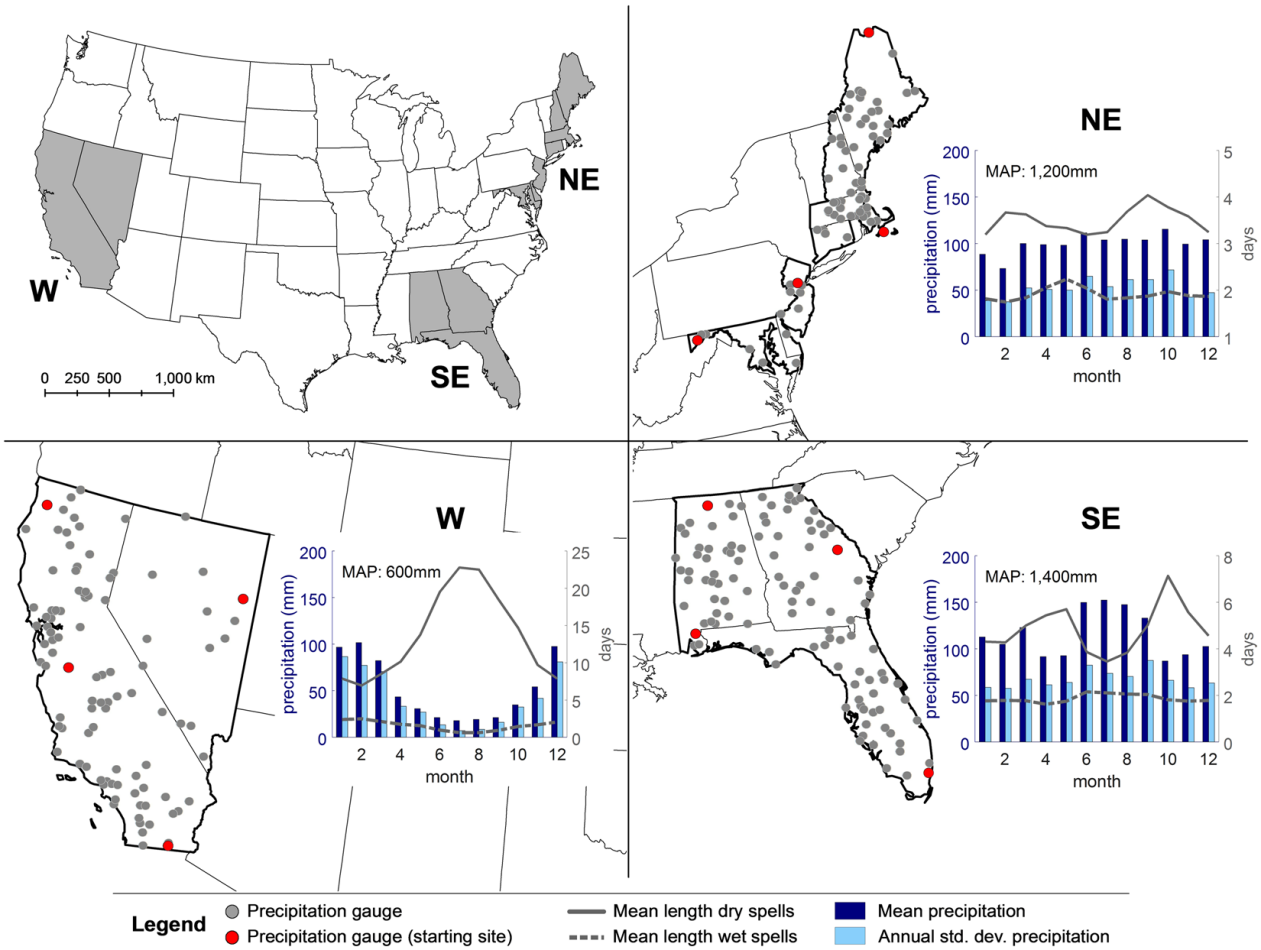
The three study areas in the North-East (NE), South-East (SE) and West (W) of the United States, including the location of all precipitation gauges available for the period 1986–2015 (grey dots), the starting sites of the experiments (see section ‘Design of the experiments’ below), plots of the mean precipitation, annual standard deviation of precipitation, mean length of dry and wet spells, averaged over all gauges for each month. The bar/line plots also contain information on the mean annual precipitation (MAP). The map was generated in ArcGIS 10.2 (http://www.esri.com/), related bar/line plots in MATLAB 2016a (http://www.mathworks.com/).

In order to fulfill the objectives of the study, a homogeneous dataset covering different climatic zones and providing a sufficiently dense observation network is needed. For these reasons, we use the dataset of daily precipitation observations available for the United States from the Global Historical Climatology Network - Daily (GHCN-Daily)^46^ for the 30-year period 1986–2015, which has been compiled by the National Climatic Data Center (NCDC) (https://www.ncdc.noaa.gov/oa/climate/ghcn-daily/). From this dataset we selected three study areas representing different climatic conditions, located in the North-East (NE), South-East (SE) and West (W) of the United States (Fig. 1).

The NE is dominated by a relatively cold climate without any dry season, with evenly distributed monthly precipitation and warmer summers. The SE is dominated by a temperate and tropical monsoon climate without any pronounced dry season and moist, hot summers. The W is dominated by an arid and semi-arid climate with frequent droughts^47^. It has a marked seasonality in precipitation and includes a temperature gradient with warmer (South) and colder regions (North). While the inter-annual variability of precipitation is more evenly distributed over the year in the NE and SE, it has a pronounced annual cycle in the W, reaching comparatively high values in the winter months. The lengths of dry and wet spells show similar annual cycles in the NE and SE with dry spells peaking in winter/spring and in the fall. Dry spells are longest in the summer in the W. For the period 1986–2015, 72 precipitation gauges with complete time series are available for the NE, 111 for the SE and 98 for the W.

### Design of the experiments

To evaluate model performance under differing conditions of data availability/scarcity, we investigated four different levels of gauge network densities (Table 1).

**Table 1:** Number of gauges for the three study areas, the four simulated precipitation gauge density scenarios referred to as ‘very high’, ‘high’, ‘medium’ and ‘low’, and the maximum extent of the networks in each scenario.

Study area/number of gauges	very high (5,200 km²/gauge)	high (11,400 km²/gauge)	medium (48,000 km²/gauge)	low (94,400 km²/gauge)	Maximum gauge network extent (km)
NE	72	32	8	4	1,173
SE	111	52	12	6	1,161
W	not available	98	23	12	1,167

The density scenarios are based on actual precipitation gauge network densities of the GHCN-Daily dataset (1986–2015) in two of the three study areas in the United States (“very high”), the average density over Europe (“high”) as well as China (“medium”) as an orientation for emerging economies, and the average density on the African continent (“low”) as an orientation for developing countries (see Figure S1 in the Supplementary material). The distribution of precipitation gauges in each scenario was conducted subjectively, aiming for equally spatially distributed networks. As each density scenario required a comparable network density for each study area, a high-density scenario could not be examined for the W.

For each density scenario, we conducted four separate experiments, each starting at one of the four so-called ‘starting sites’ (located in four different regions of each study area; see red gauges in Fig. 1). The four starting sites (i.e. four experiments starting in different regions of the study area) were introduced to capture the obviously not fully homogenous climate of each study area. Each experiment began by considering a minimum precipitation gauge network of three sites (i.e. the starting site and its two closest sites), continuously widening up the precipitation gauge network by adding the next closest precipitation gauge up to the maximum number of gauges available for each density scenario. For each precipitation gauge network, we simulated 30 different ensembles to obtain stable results, each time over the 30-year period (i.e. 900 years). In other words, for each density scenario and starting site in each study area, we performed 30 runs for a network of three gauges, 30 runs for four gauges and so on, up to 30 runs for all available gauges. For example, the total number of simulated years in the NE for the density scenario “very high” is 4 (experiments using the four starting sites) × 70 (different precipitation gauge networks between three and 72 sites) × 30 (different ensembles) × 30 (observation years) = 252,000 years. The combination of all three study areas (i.e. climates), precipitation gauge network sizes (i.e. spatial scale) and number of sites (i.e. network density) led to a total of 1,461,600 generated precipitation years.

We used the semi-parametric multi-site precipitation generator TripleM^21, 48^, which we optimized for large gauge networks to perform the experiments (see Methods). We simulated daily precipitation amounts by a pure resampling of the observations (bootstrap) to eliminate uncertainties arising from parametric precipitation sampling. A detailed description of the TripleM algorithm is available in the Methods.

## Results and Discussion

We focus on four key metrics relevant for climate change and climate change impacts studies, namely: (i) the inter-annual standard deviation of precipitation, (ii) the average maximum length of dry spells (dry periods), (iii) the mean length of dry spells, and (iv) the average maximum length of wet spells (wet periods). The intra-annual distribution of precipitation and inter-annual variability in precipitation amounts are key drivers of the functioning of terrestrial ecosystems, and hence local carbon balance, agricultural production, natural hazards such as floods and droughts, and have both direct and indirect impacts on human health and well-being^49–51^. Inter-annual variations in precipitation and temperature explain on average a third of the global crop yield variability^50^. Mean dry spells represent continuing water stress of plants^52^, while maximum dry spells (ii) are of relevancy for drought studies. Maximum wet spells (iv) influence floods and, depending on climatic conditions, have a direct impact on the prevalence of water-related vector-borne diseases, such as Chikungunya^53^ or rift valley fever^54, 55^, but also on agriculture^56^. We assessed the performance of the precipitation generator with focus on the average annual performance and on the summer (Jun, Jul, Aug) and winter (Dec, Jan, Feb) seasons separately. The precipitation generator performance was characterized as the relative error between the mean of the 30 simulations for all sites of each precipitation gauge network and the observations. Since we conducted four experiments with four starting sites in each study area, in the figures below we show the mean of these four simulations.

For a more in-depth assessment, we examined five additional standard hydrological metrics: (i) the simulated mean precipitation, (ii) the daily standard deviation of precipitation, (iii) the mean length of wet spells, (iv) the lag1 autocorrelation of precipitation occurrence as well as (v) the cross-correlation of precipitation occurrence lagged by one day as a proxy for the persistence of weather situations. The results for these metrics are reported in the Supplementary material.

### Climate and spatial scale

**Figure 2:**
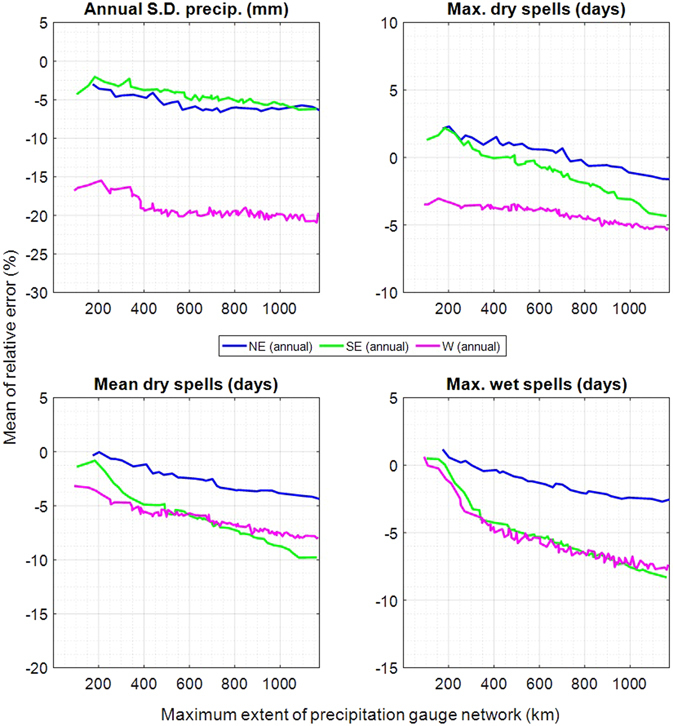
Relative error for all sites in the North-East (blue), the South-East (green) and the West (magenta) for all months. The error is plotted for four metrics against the maximum extent of each simulated gauge network. For each study area, the lines show the mean of the four simulations (using four different starting sites; see Fig. 1).

**Figure 3:**
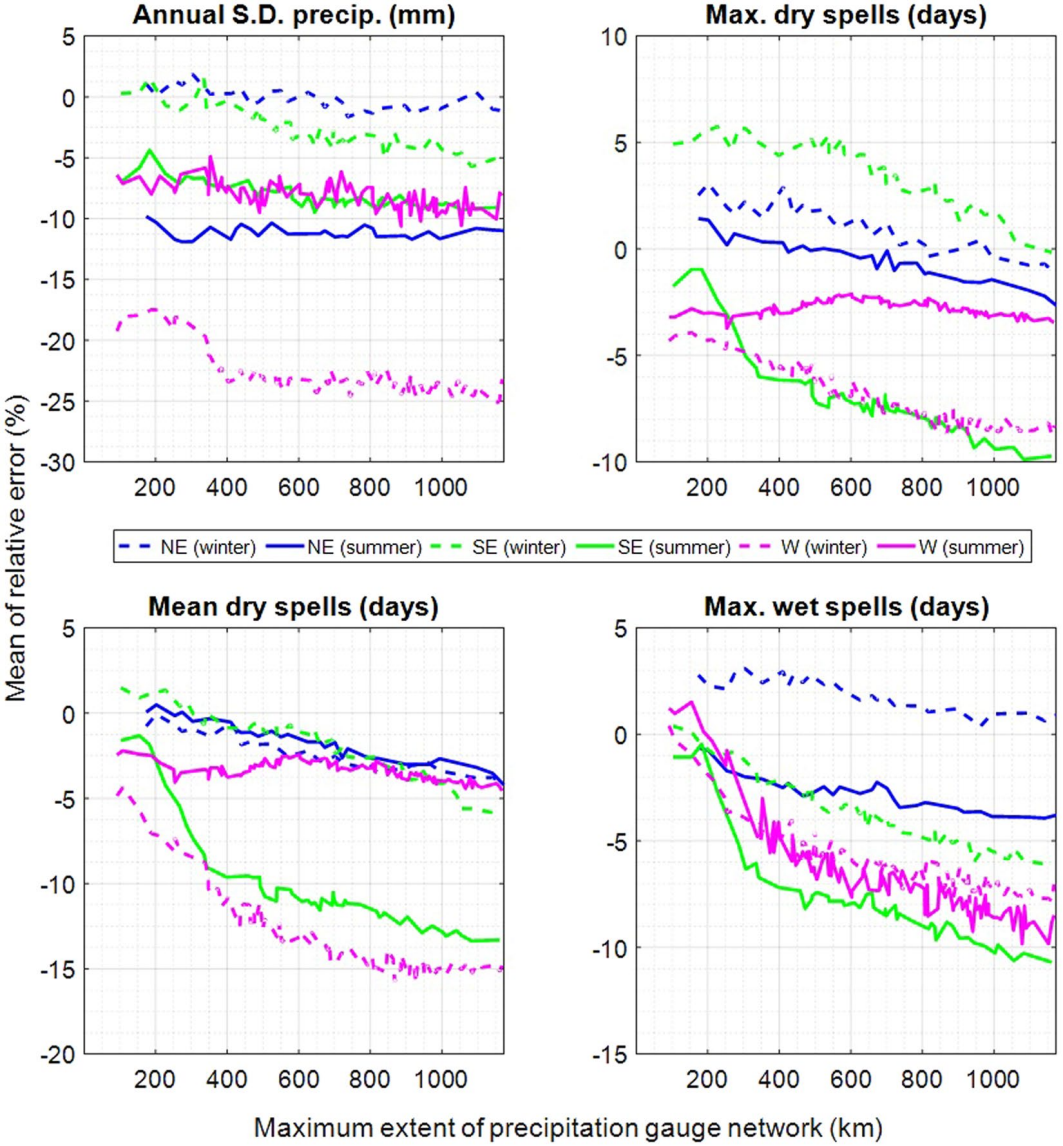
Relative error for all sites in the North-East (blue), the South-East (green) and the West (magenta) for the summer (solid lines) and winter season (dashed lines). The error is plotted for four metrics against the maximum extent of each simulated gauge network. For each study area, the lines show the mean of the four simulations (using four different starting sites; see Fig. 1).

We present the impact of the spatial scale (Figs 2 and 3) for the high density scenario (see Table 1) for the three climates. The performance generally decreases with increasing gauge network size. This is expected as in TripleM daily snapshots of precipitation occurrences are first clustered according to their similarity and then simulated based on a univariate Markov process. A larger extent means larger, less homogenous precipitation snapshots and lower performance. For the four metrics, increasing the network size increases the mean error on average by 3.7% in the NE (from 0.0% with three sites), 6.2% in the SE (from −1.0% with three sites) and 4.3% in the W (from −5.7% with three sites).

For the annual performance (Fig. 2), the inter-annual standard deviation tends to be underestimated. This is a typical phenomenon of daily weather generators, referred to as overdispersion. The underestimation is generally low for the NE and the SE and higher for the W. On average, the underestimation reaches a maximum of −6.4% in the NE (starting at −3.0% for the smallest network size) and −6.2% (starting at −4.3%) in the SE, with a slightly decreasing performance towards larger gauge networks. The underestimation in the W increases from −16.7% for three gauges to −19.6% for all sites. Daily weather generators rely on daily weather scenarios and have thus a limited capability for reproducing the inter-annual variability. The underestimation is predominately caused by the resampling approach. The bootstrap only takes into account observations and cannot generate very extreme events, which underestimates the sampling distribution, especially for small sample sizes. The latter explains the higher overdispersion in the W where precipitation events are rare. Attempts have been made to overcome this shortcoming^57–59^. For example, overdispersion could be further reduced also in TripleM-type models by introducing parametric precipitation sampling with heavy tailed distributions as suggested by Wilks^39^. However, fitting of parametric precipitation curves in dry areas may be infeasible due to the limited number of precipitation observations. Seasonal differences are shown in Fig. 3. In the NE and SE, the variability is more underestimated in the summer. The observed annual standard deviation averaged over the entire precipitation gauge network is 43.8% higher in summer than in winter in the NE and 30.1% higher in the SE. In the W, it is 8.7 times higher in winter than in summer due to the predominantly arid summer. It is an inherent property of the bootstrap that the underestimation decreases exponentially with an increasing variability of the observations. This explains why seasons with a relatively high inter-annual variability are more strongly underestimated than seasons with a relatively low variability.

The length of maximum dry spells is slightly overestimated in the NE with 2.1% for three gauges and underestimated for larger extents, reaching −1.6% at full extent (Fig. 2). The trend is similar for the SE and W, starting with an overestimation of 1.3% and underestimation of −3.5% respectively and reaching −4.3% and −5.2% at full extent. Mean dry spells are likewise least underestimated in the NE (−0.3% to −4.3%). The underestimation in the SE and W starts with −1.4% and −3.2%, reaching −9.8% and −7.9% at full extent. The bias for simulating maximum wet spells is smallest in the NE (1.2% to −2.5%). Maximum wet spells are less well reproduced in the SE and W and follow similar trends (0.5% and 0.6% to −8.3% and −7.4%).

In the NE and the SE maximum dry spells are better reproduced in winter compared to summer (Fig. 3). This is related to the persistence of weather events, expressed by the lagged cross-correlation of the precipitation occurrences, which is 6.4% higher in winter in the NE and 13.8% higher in the SE compared to summer. The clustering approach performs better when precipitation events are predominantly of frontal nature. The convective systems that are common in summer are more variable, with smaller scales in time and space, thus leading to more distinctive precipitation patterns and reducing the clustering performance. The performance for mean dry spells is similar with almost equal performance in summer and winter in the NE.

Performance differences between the NE and the SE are related to the strong impact of convective systems in the SE, particularly in summer: Florida is the state in the United States with the highest thunderstorm activity^60, 61^. Precipitation contribution of tropical cyclones to the seasonal precipitation totals can reach up to 20% in the coastal regions, with comparatively high inter-annual variabilities depending on whether a year has hurricane observations or not^62^. According to the International Best Track Archive for Climate Stewardship IBTrACS^63^ (release version v03r09), the South-East study area as presented in this research has been hit by 23 named and three unnamed tropical cyclones between 1986 and 2015 in the summer season. Conversely, precipitation in the NE is predominately of frontal nature. According to a study by Hawcroft *et al*.^64^ using two different reanalysis datasets the contribution of extratropical cyclones to the total precipitation in the NE study area reaches over 80% in the winter season and over 65% in the summer season with uncertainties of up to about 20% depending on the reanalysis dataset under investigation. In the W, the precipitation climatology is much more complex, with a pronounced spatial heterogeneity of precipitation with a large impact of smaller-scale climatic controls in the mountainous areas^65^. The region is also strongly influenced by the El Niño–Southern Oscillation (ENSO). In the Great Basin, which covers most of the Western study area except for California, above normal precipitation between October and March is predominately associated to ENSO years^66^. The inter-annual variability is also linked to ENSO^67^. The mountain ranges of the Sierra Nevada in California receive high precipitation amounts due to orographic effects, which also explain the dry conditions in the Great Basin because of a rain shadow effect. The lagged cross-correlation of observed precipitation occurrences (i.e. weather persistence) is three times higher in winter than summer, due to the dominant influence of midlatitudinal synoptic-scale storms^68, 69^. The still better performance for dry spells in the W in summer is related to the arid summer (recorded precipitation on only 4.3% of all days), making a pronounced underestimation of dry spells unlikely. The performance for maximum wet spells in the NE and in the SE is similar to the performance in regard to dry spells. The performance is better during winter with higher persistence of weather events. Maximum wet spells are equally reproduced in both seasons in the W. The 90% confidence intervals (see Supplementary material) show similar spreads across seasons and study areas. The most significant differences are related to the W: For summer, confidence intervals are significantly wider for the majority of metrics, which is related to the low number of precipitation days. The results for the medium and low gauge density scenarios (Table 1, not shown here) showed comparable results.

### Network density and spatial scale

**Figure 4:**
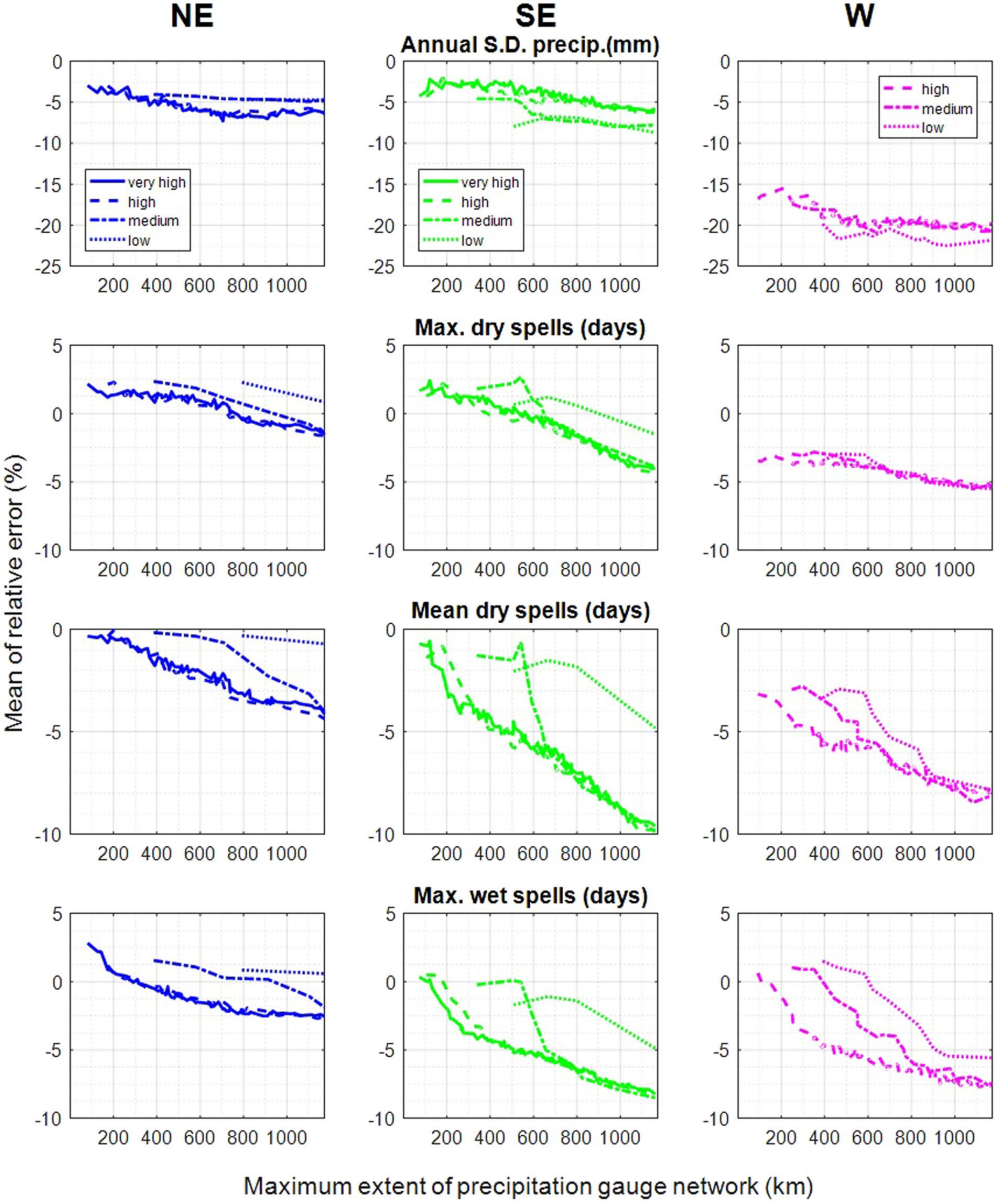
Relative error for all sites in the North-East (NE), the South-East (SE) and the West (W) for all months. The error is plotted for four metrics against the maximum extent of each simulated gauge network and density scenario. For each study area and scenario, the lines show the mean of the four simulations (using four different starting sites; see Fig. 1). The solid line represents the results for the very-high gauge network density (not available for the West), the dashed line for the high-density, the dash-dot line for the medium density and the dotted line for the low gauge density scenario.

The gauge network density impacts the performance. Here, for all available density scenarios (Table 1), we focus on the annual performance only (Fig. 4), but seasonal performances are comparable.

Deviations between network densities can be encountered. For the majority of the metrics, the model bias decreases with a reduced network density, with differences between about one to five percent, depending on the study area and maximum extent. However, low density does not always mean better performance, primarily for the inter-annual standard deviation of precipitation in the SE and W. To reach the same fixed duplication rate of observations, fewer clusters of daily precipitation snapshots are required for small networks. Thus, the clusters represent weather situations less well, which effectively reduces the model performance. The opposite applies to dry and wet spells where the bias decreases with a reduced network density. The most pronounced differences can be recognized for maximum and mean dry spells in the SE, and maximum wet spells in the SE and in the W. The phenomenon is likewise caused by the clustering algorithm. A smaller number of gauges leads to a better distinction between the clustered daily precipitation snapshots and therefore higher similarity within these clusters, which improves the performance. Deviations are higher in the less homogenous climates of the SE and W. The slightly better performance may give the impression that a lower dense gauge network may likewise be preferable, but (i) differences in the performance do not exceed differences of one to five percent and (ii) most applications require the interpolation of the simulated precipitation patterns, where a high number of stations is desirable.

## Conclusion

This study is a first step towards overcoming the fragmented, eclectic knowledge in stochastic generation of precipitation patterns and is thereby a call for testing multiple, and possibly publicly available, model codes across different climate types, spatial scales and network densities. The comparison of 30-year long observed daily precipitation patterns with generated precipitation across three different climates shows a general adequate agreement when considering relatively small regions, although key metrics such as dry or wet spells are often underestimated. Larger spatial scales lead to reduced performance in reproducing the observations. The simulations are less biased in wet temperate climates than in dry climates. Seasons and locations that are dominated by frontal precipitation are better reproduced than seasons with a more pronounced impact of convective systems. This explains the different performance obtained in the temperate North-East and subtropical South-East. Seasons with a higher inter-annual variability of precipitation are less well reproduced, as demonstrated with the Western study area, which is influenced by ENSO.

In this research, we focused on the current climate. There are different approaches to parameterize precipitation generators to simulate climate change, for example by altering the precipitation values using output from climate models as for example suggested by Turkington *et al*.^13^. However, as the pure alteration of the precipitation amounts ignores potential future changes in dry and wet spells, another promising avenue could be to condition the clustering of the daily precipitation snapshots in the TripleM model to the distribution of current and future circulation patterns to incorporate changes of dry spells, wet spells and also in the autocorrelation of precipitation. Simulating climate change with weather generators however has inherent limitations in regard to decadal variabilities and long-term trends. The consideration of other climate types beyond the three of this study would be another interesting topic for investigation.

The development of common evaluation standards as for instance information on relative errors for better comparability is highly desirable. Additional comparative studies particularly in countries with lower network densities (Figure S1, Supplementary material) would be useful to validate the findings of this study. Further, the proposed methodology should be complemented to enable simulating projected precipitation patterns that can be used for climate change impact studies, ideally in the developing world, where impacts of climate change are often most significant. At this point in time, facing numerous published types of algorithms, eclectic case studies, a very limited number of transparent publicly available source codes and a lack of common evaluation standards, the full potential of stochastic multi-site weather generation remains unclear. The issue magnifies when different model types are parameterized for simulating future climate scenarios. We made a first step towards closing this gap by demonstrating that – if there is awareness and knowledge of stochastic approaches and model type specific opportunities and shortcomings – stochastic multi-site precipitation generation has the potential to support a variety of societally and ecologically relevant issues in different climates, at different spatial scales and under differing conditions of data availability.

## Methods

The reduced complexity multi-site precipitation generator TripleM (Multisite Markov Model) applied here works as follows: First, daily snapshots of the precipitation occurrences (i.e. catchment-wide precipitation patterns) are clustered according to their similarity. The model uses the non-hierarchical k-means clustering method^70, 71^ and the hamming distance (equation (1)).

1 $$distance(x,y)=\frac{1}{p}\,\,\sum _{j=1}^{p}I\{{x}_{j}\ne {y}_{j}\},$$

where *I* is the indicator function.

In the original version of TripleM^48^, the k-means clustering was applied to daily snapshots of precipitation amounts that were first standardized using the z-score transformation in order to take into account the heteroscedastic nature of the precipitation. This led to a satisfying performance in a comparatively small Alpine precipitation gauge network not exceeding a maximum distance between sites of about 150 km. For this research, we ran multiple experiments with different clustering methods and it turned out that the performance increases significantly for large gauge networks when applying the hamming distance to binary precipitation occurrences.

Second, the clustered occurrence vectors are simulated with a Markov process (equation (2)), where the transition probabilities depend on *m* previous days, i.e.,

2 $$\mathrm{PR}\{{X}_{t+1}|{X}_{t},{X}_{t-1},{X}_{t-2},\ldots ,{X}_{1}\}=\mathrm{PR}\{{X}_{t+1}|{X}_{t},{X}_{t-1},\ldots ,{X}_{t-m}\}\,\mathrm{with}\,m < t-1$$

**Figure 5:**
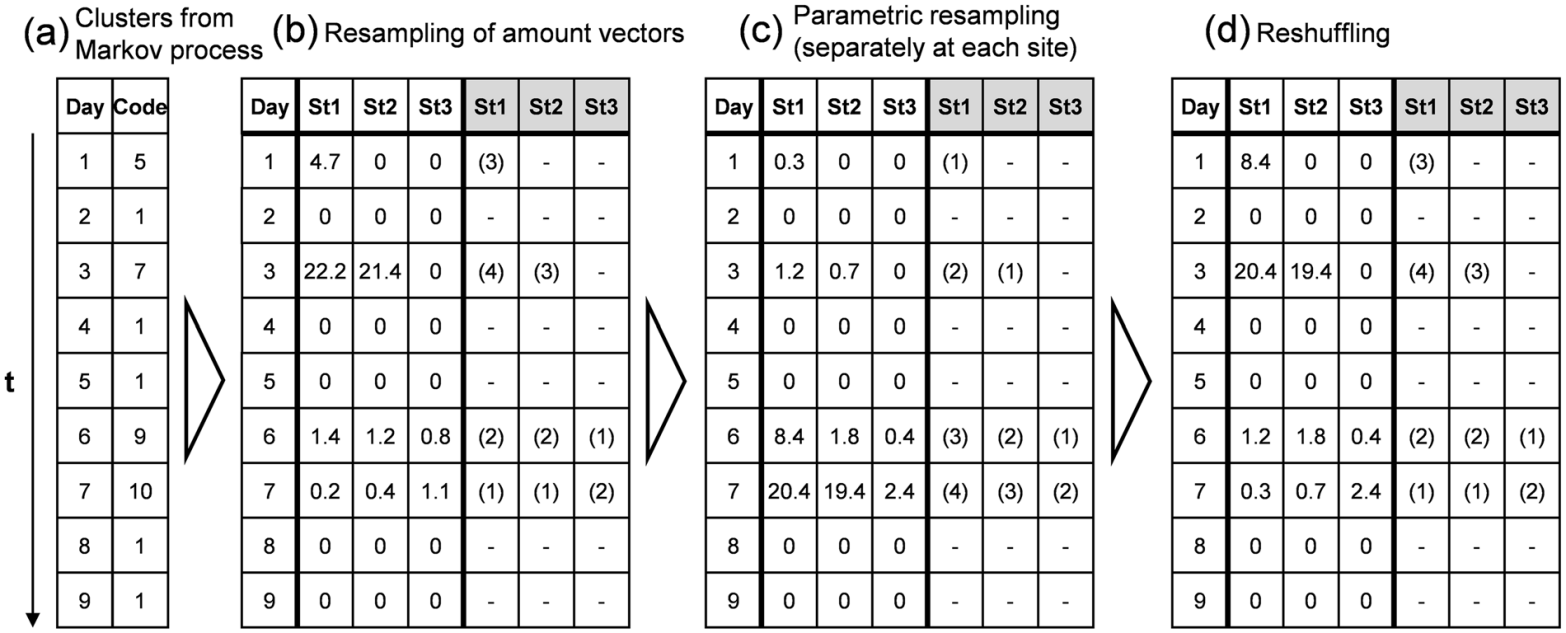
Key steps of precipitation generation in TripleM after clustering of the daily precipitation snapshots and Markov simulation (**a**), including resampling of amount vectors (**b**), parametric sampling (**c**) and reshuffling (**d**).

Once the synthetic time series of clusters are simulated, each cluster is replaced by a random amount vector (i.e. daily snapshot of precipitation amounts) belonging to the same cluster. In a last step, which is optional, the model introduces sampling of parametric precipitation amounts in combination with an adapted version of a resampling approach by Clark *et al*.^41^, to account for unobserved precipitation extremes. The method is shown in Fig. 5, using a hypothetical example of three sites and a ten states Markov chain: After generating synthetic time series of clusters using the Markov process (a), amount vectors are randomly drawn from all observations that fit the corresponding cluster (b). Following this, synthetic precipitation amounts are sampled independently for each site from parametric curves (c) optionally using correlated uniform random numbers from a Cholesky decomposition^72^. The use of correlated random numbers avoids the generation of significantly different precipitation amounts across sites, which becomes increasingly important when generating short synthetic time series. In the last step (d), the parametric precipitation amounts are reshuffled according to the original ranks after the resampling in step (b), to maintain the inter-site correlations.

**Figure 6:**
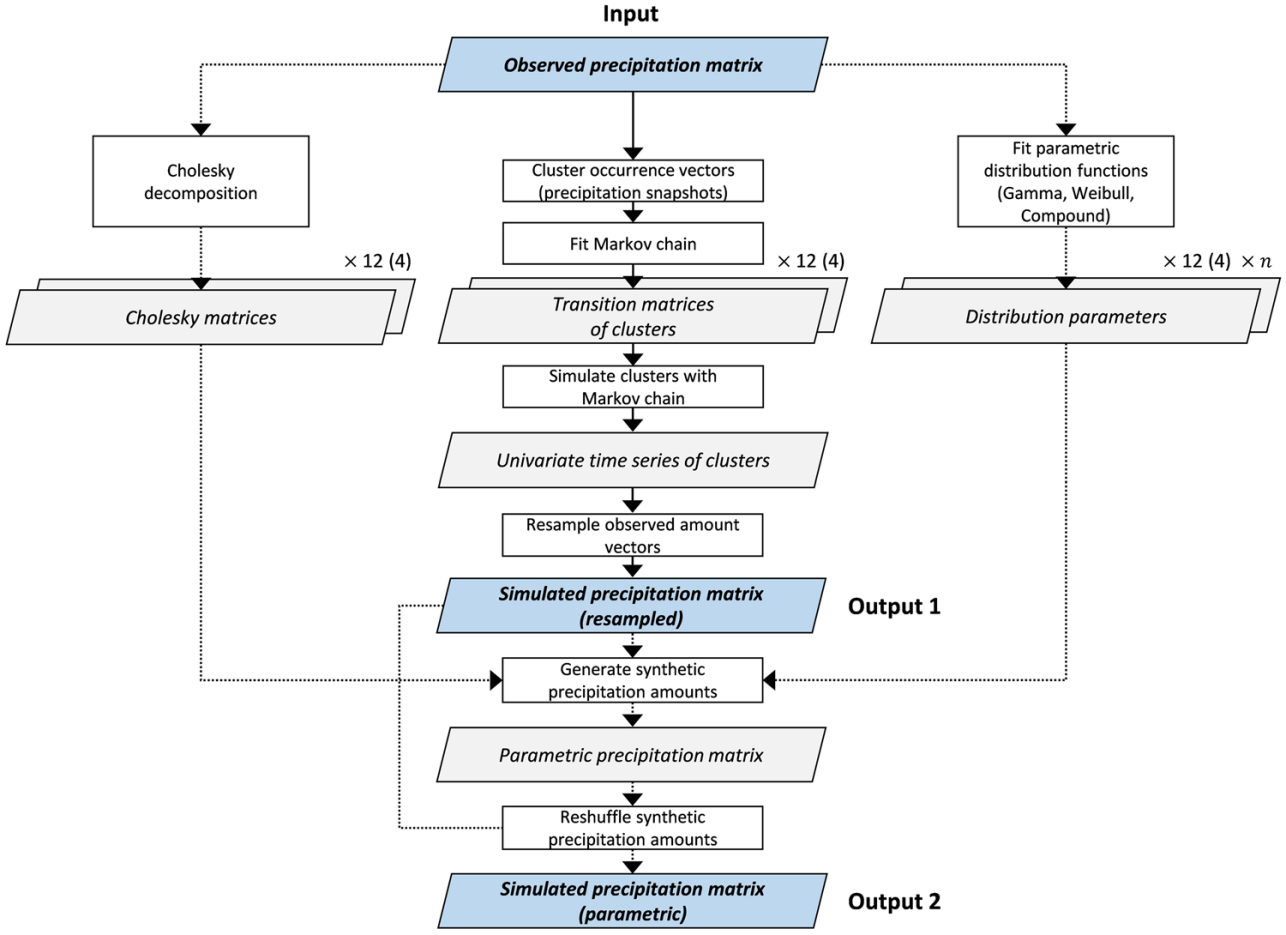
Schematic flow diagram of the TripleM precipitation generator. TripleM can be used as a bootstrap model (Output 1) and a parametric precipitation model (Output 2). Parallelograms represent time series or variables, boxes represent methods. Blue parallelograms represent input and output data. Cholesky matrices and transition matrices are either derived monthly (12) or seasonally (4). The parametric distribution parameters are either derived monthly (12) or seasonally (4) for the number of gauges simulated (n).

The entire simulation process can be depicted from Fig. 6.

In its most simplistic setup (resampling i.e. bootstrap without parametric sampling of precipitation amounts as applied in this study), TripleM has two key parameters the user has to define: the duplication rate and the order of the Markov chain. As for the duplication rate, an inherent characteristic of TripleM is that the clustering approach will duplicate parts of the time series: A higher number of clusters will generally improve the reproduction of various metrics of the observations such as the precipitation autocorrelation, but result in duplicated observations in the simulations, especially in large station networks. Here and elsewhere^48^, a maximum duplication rate of only 1% produced satisfying results. Higher duplications rates increase the computational costs. The second key parameter is the order of the Markov chain used. In this study, a one-order Markov chain was used. For larger observation networks, it is recommended not to increase the order due to the exponentially growing state-space related to higher orders.

Another model specific characteristic is the reproduction of inter-site correlations. If long synthetic time series are generated in combination with parametric precipitation sampling, inter-site correlations are better reproduced. This is caused by the reshuffling method. With long synthetic time series, the pool of parametric precipitation amounts becomes more similar to the resampled precipitation amounts. In TripleM, the reshuffling is conducted over all generated years separately for all months or seasons depending on the chosen model setup. The choice of parametric models for the synthetic precipitation amounts is another influencing factor in general, which has been discussed in the past^36, 73, 74^ and is not specific to TripleM. The MATLAB code offers the Gamma distribution, the Weibull distribution or a compound distribution of the Weibull distribution for lower and a Generalized Pareto distribution for higher and extreme precipitation amounts with a user-defined threshold between both curves.

TripleM offers monthly and seasonal setups. All steps, including the clustering of amount vectors, the fitting of the Markov chains, the simulation of the Markov process and the reshuffling of parametric precipitation amounts, can either be run monthly or seasonally. In this study we used a monthly setup.

### Code and data availability

The MATLAB source code of TripleM, a user manual and a training dataset are available from the github page, https://github.com/KBreinl/TripleM. The data used in this paper are available from the NOAA websites.

## Electronic supplementary material

Supplementary Material

## References

[1] Aerts JCJH, Botzen WJW (2011). Climate change impacts on pricing long-term flood insurance: A comprehensive study for the Netherlands. Global Environ Chang.

[2] Van Loon AF (2016). Drought in the Anthropocene. Nat Geosci.

[3] AghaKouchak A (2011). Geometrical Characterization of Precipitation Patterns. J Hydrometeorol.

[4] Schwartz CS (2010). Toward Improved Convection-Allowing Ensembles: Model Physics Sensitivities and Optimizing Probabilistic Guidance with Small Ensemble Membership. Weather Forecast.

[5] Bray M (2011). Rainfall uncertainty for extreme events in NWP downscaling model. Hydrol Process.

[6] Ciais P (2005). Europe-wide reduction in primary productivity caused by the heat and drought in 2003. Nature.

[7] Piao SL (2008). Net carbon dioxide losses of northern ecosystems in response to autumn warming. Nature.

[8] Burton A (2010). Downscaling transient climate change using a Neyman-Scott Rectangular Pulses stochastic rainfall model. J Hydrol.

[9] Feddersen H, Andersen U (2005). A method for statistical downscaling of seasonal ensemble predictions. Tellus A.

[10] Palutikof JP (2002). Generating rainfall and temperature scenarios at multiple sites: Examples from the Mediterranean. J Climate.

[11] Forsythe N (2014). Application of a stochastic weather generator to assess climate change impacts in a semi-arid climate: The Upper Indus Basin. J Hydrol.

[12] Jones, P. D. *et al*. Downscaling regional climate model outputs for the Caribbean using a weather generator. *Int J Climatol*, **36**, 4141–4163 (2016).

[13] Turkington, T. *et al*. A new flood type classification method for use in climate change impact studies. *Weather and Climate Extremes* **14**, 1–16 (2016).

[14] Trnka M (2014). Adverse weather conditions for European wheat production will become more frequent with climate change. Nat Clim Change.

[15] Holding S (2016). Groundwater vulnerability on small islands. Nat Clim Change.

[16] Breinl, K. *et al*. A joint modelling framework for daily extremes of river discharge and precipitation in urban areas. *Journal of Flood Risk Management* **10**, 97–114 (2017).

[17] Qin XS, Lu Y (2014). Study of Climate Change Impact on Flood Frequencies: A Combined Weather Generator and Hydrological Modeling Approach. J Hydrometeorol.

[18] Khazaei MR (2012). Assessment of climate change impact on floods using weather generator and continuous rainfall-runoff model. Int J Climatol.

[19] Harris CNP (2014). The use of probabilistic weather generator information for climate change adaptation in the UK water sector. Meteorol Appl.

[20] Leander R, Buishand TA (2009). A daily weather generator based on a two-stage resampling algorithm. J Hydrol.

[21] Breinl K (2016). Driving a lumped hydrological model with precipitation output from weather generators of different complexity. Hydrolog Sci J.

[22] Hansen JW, Ines AVM (2005). Stochastic disaggregation of monthly rainfall data for crop simulation studies. Agr Forest Meteorol.

[23] Greene AM (2015). A climate generator for agricultural planning in southeastern South America. Agr Forest Meteorol.

[24] Mearns LO (1997). Mean and variance change in climate scenarios: Methods, agricultural applications, and measures of uncertainty. Clim Change.

[25] Stevens, T. & Madani, K. Future climate impacts on maize farming and food security in Malawi. *Scientific Reports* **6** (2016). 10.1038/srep36241 PMC5099946 27824092

[26] Semenov, M. A. & Shewry, P. R. Modelling predicts that heat stress, not drought, will increase vulnerability of wheat in Europe. *Scientific Reports* **1** (2011). 10.1038/srep00066 PMC3216553 22355585

[27] Charron, D. F. *et al*. Links Between Climate, Water And Waterborne Illness, and Projected Impacts of Climate Change. *Health Canada* (2005).

[28] Morin CW, Comrie AC (2013). Regional and seasonal response of a West Nile virus vector to climate change. P Natl Acad Sci USA.

[29] Ogden NH (2006). Climate change and the potential for range expansion of the Lyme disease vector Ixodes scapularis in Canada. Int J Parasitol.

[30] Clare, F. C. *et al*. Climate forcing of an emerging pathogenic fungus across a montane multi-host community. *Philosophical Transactions of the Royal Society B: Biological Sciences* **371** (2016). 10.1098/rstb.2015.0454 PMC5095533 28080980

[31] Baigorria GA, Jones JW (2010). GiST: A Stochastic Model for Generating Spatially and Temporally Correlated Daily Rainfall Data. J Climate.

[32] Bardossy A, Pegram GGS (2009). Copula based multisite model for daily precipitation simulation. Hydrol Earth Syst Sc.

[33] Serinaldi, F. A multisite daily rainfall generator driven by bivariate copula-based mixed distributions. *J Geophys Res-Atmos* **114** (2009).

[34] Brissette FP (2007). Efficient stochastic generation of multi-site synthetic precipitation data. J Hydrol.

[35] Serinaldi F (2009). Copula-based mixed models for bivariate rainfall data: an empirical study in regression perspective. Stoch Env Res Risk A.

[36] Breinl K (2013). Stochastic generation of multi-site daily precipitation for applications in risk management. J Hydrol.

[37] Khazaei M (2013). A new daily weather generator to preserve extremes and low-frequency variability. Clim Change.

[38] Leander R, Buishand TA (2007). Resampling of regional climate model output for the simulation of extreme river flows. J Hydrol.

[39] Wilks DS (1998). Multisite generalization of a daily stochastic precipitation generation model. J Hydrol.

[40] Rayner D (2016). A multi-state weather generator for daily precipitation for the Torne River basin, northern Sweden/western Finland. Advances in Climate Change Research.

[41] Clark, M. P. *et al*. A resampling procedure for generating conditioned daily weather sequences. *Water Resour Res* **40** (2004).

[42] Mehrotra R (2006). A comparison of three stochastic multi-site precipitation occurrence generators. J Hydrol.

[43] Mehrotra R, Sharma A (2007). A semi-parametric model for stochastic generation of multi-site daily rainfall exhibiting low-frequency variability. J Hydrol.

[44] Apel H (2016). Combined fluvial and pluvial urban flood hazard analysis: concept development and application to Can Tho city, Mekong Delta, Vietnam. Nat. Hazards Earth Syst. Sci..

[45] Ailliot P (2015). Stochastic weather generators: an overview of weather type models. J Soc Fr Statistique.

[46] Menne MJ (2012). An Overview of the Global Historical Climatology Network-Daily Database. J Atmos Ocean Tech.

[47] AghaKouchak A (2015). Water and climate: Recognize anthropogenic drought. Nature.

[48] Breinl K (2015). Simulating daily precipitation and temperature: a weather generation framework for assessing hydrometeorological hazards. Meteorol Appl.

[49] Knapp AK, Smith MD (2001). Variation among biomes in temporal dynamics of aboveground primary production. Science.

[50] Ray, D. K. *et al*. Climate variation explains a third of global crop yield variability. *Nat Commun* **6** (2015). 10.1038/ncomms6989 PMC4354156 25609225

[51] Porporato, A. *et al*. Superstatistics of hydro-climatic fluctuations and interannual ecosystem productivity. *Geophys Res Lett* **33** (2006).

[52] Frank DA (2015). Effects of climate extremes on the terrestrial carbon cycle: concepts, processes and potential future impacts. Global Change Biol.

[53] Fischer, D. *et al*. Climate change effects on Chikungunya transmission in Europe: geospatial analysis of vector’s climatic suitability and virus’ temperature requirements. *Int J Health Geogr* **12** (2013). 10.1186/1476-072X-12-51 PMC3834102 24219507

[54] Linthicum KJ (1999). Climate and satellite indicators to forecast Rift Valley fever epidemics in Kenya. Science.

[55] Taylor D (2016). Environmental change and Rift Valley fever in eastern Africa: projecting beyond HEALTHY FUTURES. Geospatial Health.

[56] Lobell DB (2011). Climate extremes in California agriculture. Clim Change.

[57] Katz RW, Parlange MB (1998). Overdispersion phenomenon in stochastic modeling of precipitation. J Climate.

[58] Kim Y (2012). Reducing overdispersion in stochastic weather generators using a generalized linear modeling approach. Climate Res.

[59] Chen J (2010). A daily stochastic weather generator for preserving low-frequency of climate variability. J Hydrol.

[60] Orville RE, Huffines GR (2001). Cloud-to-ground lightning in the United States: NLDN results in the first decade, 1989–98. Mon Weather Rev.

[61] Hodanish S (1997). A 10-yr monthly lightning climatology of Florida: 1986–95. Weather Forecast.

[62] Prat OP, Nelson BR (2013). Precipitation Contribution of Tropical Cyclones in the Southeastern United States from 1998 to 2009 Using TRMM Satellite Data. J Climate.

[63] Knapp KR (2010). The International Best Track Archive for Climate Stewardship (Ibtracs) Unifying Tropical Cyclone Data. B Am Meteorol Soc.

[64] Hawcroft, M. K. *et al*. How much Northern Hemisphere precipitation is associated with extratropical cyclones? *Geophys Res Lett* **39** (2012).

[65] Mock CJ (1996). Climatic Controls and Spatial Variations of Precipitation in the Western United States. J Climate.

[66] Ropelewski CF, Halpert MS (1987). Global and Regional Scale Precipitation Patterns Associated with the El-Nino Southern Oscillation. Mon Weather Rev.

[67] Rajagopalan B, Lall U (1998). Interannual variability in western US precipitation. J Hydrol.

[68] Cayan DR, Roads JO (1984). Local Relationships between United-States West-Coast Precipitation and Monthly Mean Circulation Parameters. Mon Weather Rev.

[69] Lareau NP, Horel JD (2012). The Climatology of Synoptic-Scale Ascent over Western North America: A Perspective on Storm Tracks. Mon Weather Rev.

[70] Hartigan, J. A. *Clustering Algorithms*. (Wiley, 1975).

[71] Hartigan JA, Wong MA (1979). Algorithm AS 136: A K-Means Clustering Algorithm. J Roy Stat Soc C.

[72] Watkins, D. S. *Fundamentals of matrix computations*. 3rd edn, (Wiley, 2010).

[73] Papalexiou SM (2013). How extreme is extreme? An assessment of daily rainfall distribution tails. Hydrol Earth Syst Sc.

[74] Vlcek O, Huth R (2009). Is daily precipitation Gamma-distributed? Adverse effects of an incorrect use of the Kolmogorov-Smirnov test. Atmos Res.

