# Supplementary material for: Can weather generation capture precipitation patterns across different climates, spatial scales and under data scarcity?
Source: Sci Rep. 2017 Jul 14;7:5449. doi: 10.1038/s41598-017-05822-y (PMC5511179; doi:10.1038/s41598-017-05822-y)
Supplement: Supplementary file 1 — Supplementary Material [file 41598_2017_5822_MOESM1_ESM.pdf]

Can weather generation capture precipitation patterns across different climates, spatial scales and under data scarcity?

Korbinian Breinl[1]*, Giuliano Di Baldassarre[1], Marc Girons Lopez[2], Michael Hagenlocher[3], Giulia Vico[4], and Anna Rutgersson[1]

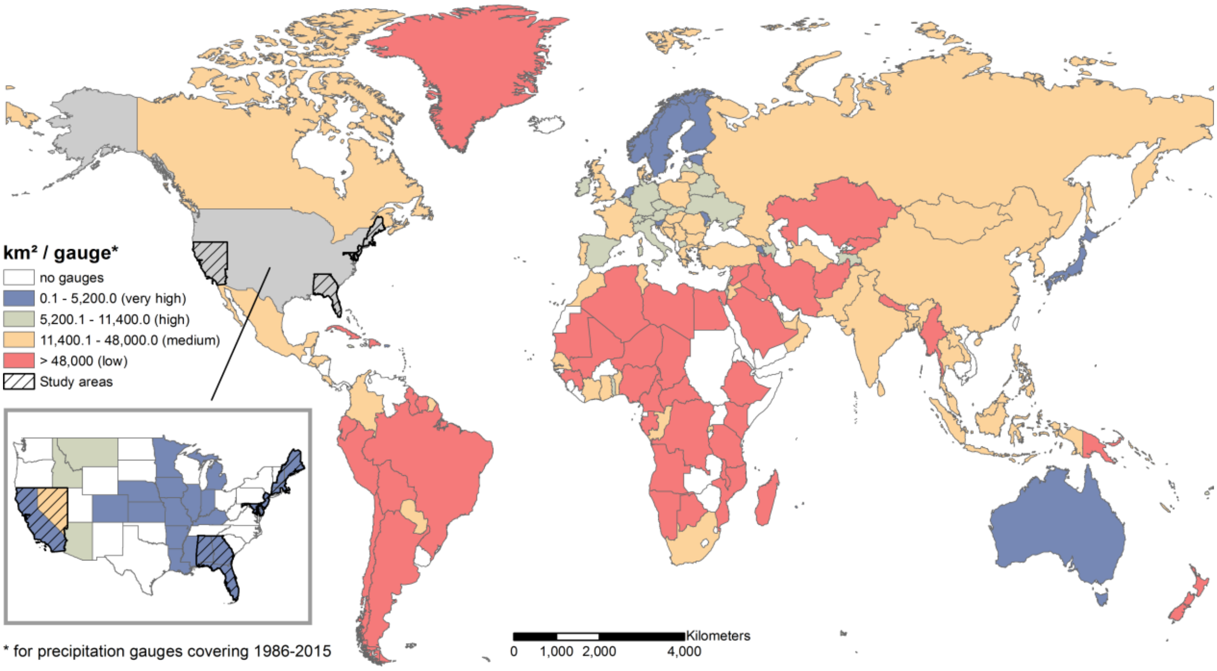

Figure S1. Averaged inverse of the precipitation gauge density per country (US: states) for the global GHCN-Daily dataset based on the precipitation gauges available for the period 1986-2015. The four density classes refer to the four gauge density scenarios (Table 1 in the main body of the text) used in the study areas of the United States. The map was generated in ArcGIS 10.2 (http://www.esri.com/).

[1] Department of Earth Sciences, Uppsala University, Villavägen 16, 75236 Uppsala, Sweden
[2] Department of Geography, University of Zurich, Winterthurerstrasse 190, 8057 Zurich, Switzerland
[3] Institute for Environment and Human Security, United Nations University (UNU-EHS), UN Campus, Platz der Vereinten Nationen 1, 53113 Bonn, Germany
[4] Department of Crop Production Ecology, Swedish University of Agricultural Sciences, Ulls väg 16, 75007 Uppsala, Sweden
* korbinian.breinl@geo.uu.se

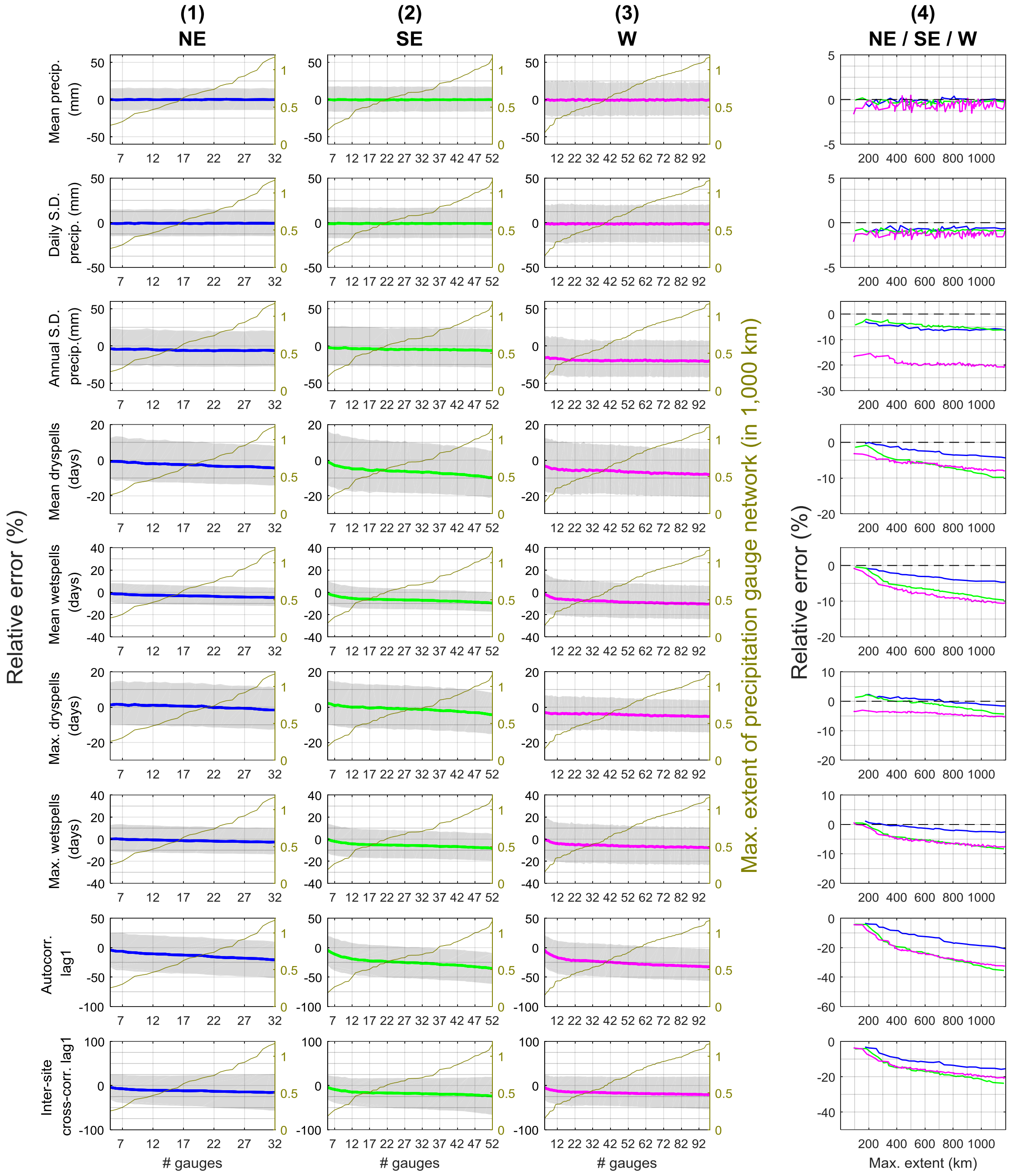

Figure S2. Simulation results for all three study areas (North-East 'NE', South-East 'SE' and West 'W') of the high density scenario 1 for all nine examined metrics and all months. The coloured lines in the columns 1 to 3 represent the mean of the relative error against the number of sites, the grey areas represent the 5th and 95th percentiles. The second y-axis in the columns 1 to 3 represents the maximum extent of each gauge network (mean of the four simulations). Column 4 shows the mean of the relative error combined for all three study areas for better comparability. The figure shows the mean of all four simulations using the four starting sites in each study area.

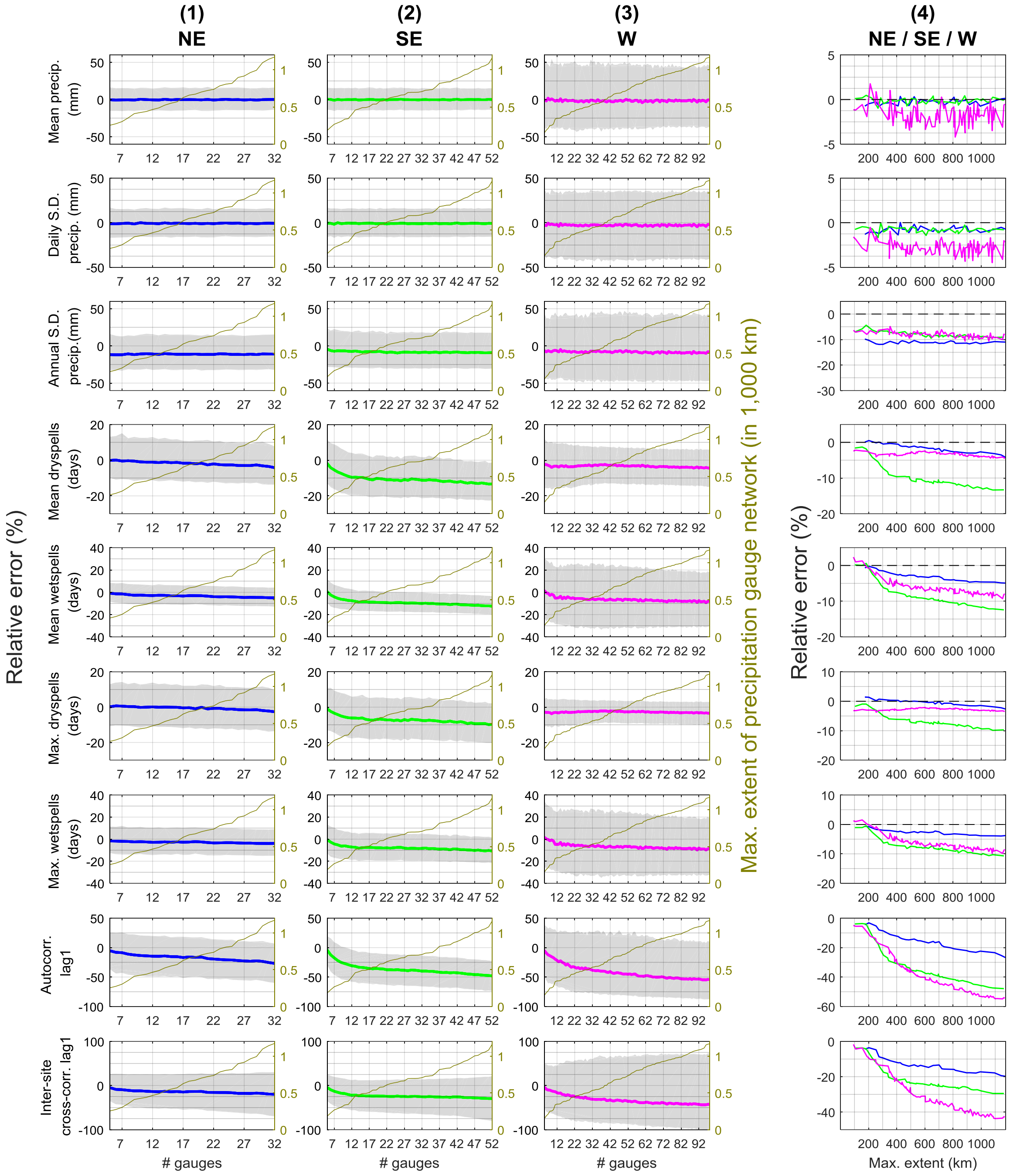

Figure S3. Simulation results for all three study areas (North-East 'NE', South-East 'SE' and West 'W') of the high density scenario 1 for all nine examined metrics and the summer season (June, July, August). The coloured lines in the columns 1 to 3 represent the mean of the relative error against the number of sites, the grey areas represent the 5th and 95th percentiles. The second y-axis in the columns 1 to 3 represents the maximum extent of each gauge network (mean of the four simulations). Column 4 shows the mean of the relative error combined for all three study areas for better comparability. The figure shows the mean of all four simulations using the four starting sites in each study area.

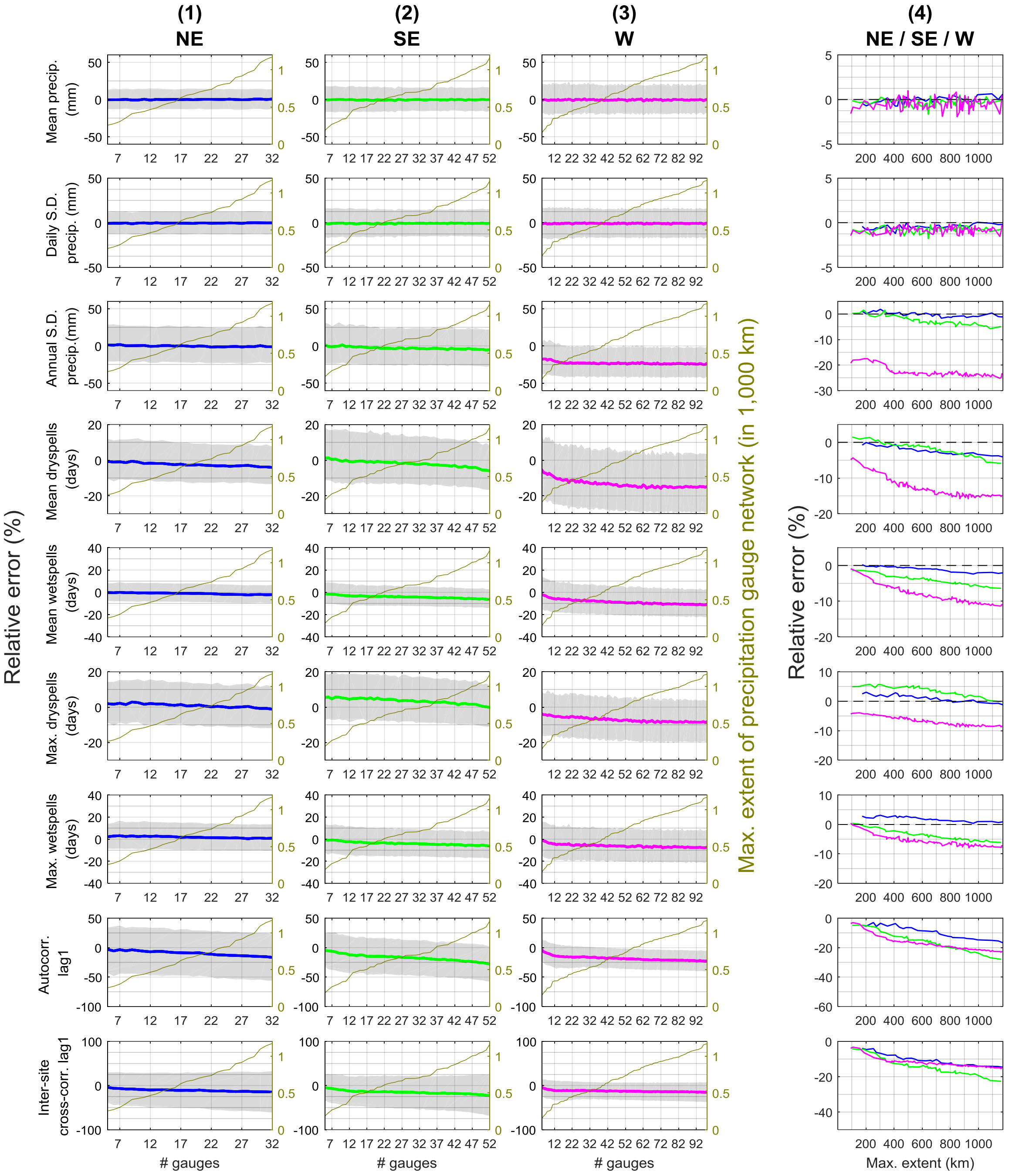

Figure S4. Simulation results for all three study areas (North-East 'NE', South-East 'SE' and West 'W') of the high density scenario 1 for all nine examined metrics and the winter season (December, January, February). The coloured lines in the columns 1 to 3 represent the mean of the relative error against the number of sites, the grey areas represent the 5th and 95th percentiles. The second y-axis in the columns 1 to 3 represents the maximum extent of each gauge network (mean of the four simulations). Column 4 shows the mean of the relative error combined for all three study areas for better comparability. The figure shows the mean of all four simulations using the four starting sites in each study area.

We examined five other metrics, i.e. (i) the simulated mean precipitation, (ii) the daily standard deviation of precipitation, (iii) the mean length of wet spells, (iv) the lag1 precipitation autocorrelation as well as (v) the cross-correlation lagged by one day as a proxy for the persistence of weather situations. Detailed plots of the results for the high-density gauge density scenario (annual, summer and winter season) are provided in Figure S2 (annual), Figure S3 (summer) and Figure S4 (winter). Here, we want to highlight the tendency of the approach to (on annual basis, Figure S2) slightly underestimate the mean precipitation in the West by 0.7%, which is related to the resampling approach, especially when the annual precipitation occurs on only a few days. A small underestimation can also be detected for the North-East (-0.1%) and South-East (-0.3%). Accordingly, the resampling approach leads to a small underestimation of the daily standard deviation of -0.7% in the North-East, -0.9% in the South-East and -1.2% in the West. This shortcoming can be addressed by introducing parametric precipitation sampling, which however requires a sufficient number of precipitation observations. Test simulations with a 10% instead of a 1% duplication rate reduced the mean error over all nine metrics by only 0.3% (North-East), 0.7% (South-East) and 1.2% (West) but increased the run times by an average factor of 2.1.
